# *Cryptococcus* and Beyond—Inositol Utilization and Its Implications for the Emergence of Fungal Virulence

**DOI:** 10.1371/journal.ppat.1002869

**Published:** 2012-09-13

**Authors:** Chaoyang Xue

**Affiliations:** 1 Public Health Research Institute, University of Medicine and Dentistry of New Jersey, Newark, New Jersey, United States of America; 2 Department of Microbiology and Molecular Genetics, University of Medicine and Dentistry of New Jersey, Newark, New Jersey, United States of America; Duke University Medical Center, United States of America

There are over one million fungal species in nature, but only a handful of them cause human diseases. A variety of distinct factors aid the virulence of fungi in their transition from environmental reservoirs to mammals. One important factor is their ability to acquire nutrients efficiently so that they can survive and thrive in a nutrient-limiting host environment. The human fungal pathogen *Cryptococcus neoformans (C. neoformans)* is the most common cause of fungal meningitis, yet the mechanisms of *Cryptococcus* neurotropism remain poorly understood. Recent studies have revealed that *Cryptococcus* has evolved sophisticated acquisition systems to utilize the carbohydrate inositol both in plant niches and in human brains, where abundant inositol is available. Inositol utilization in *Cryptococcus* and its likely contribution to *Cryptococcus* virulence may represent one example of a common trait for the emergence of pathogens from environmental reservoirs.

## *Cryptococcus* Can Undergo Sexual Reproduction by Utilizing Inositol from Plants

*C. neoformans* and its sibling species *Cryptococcus gattii (C. gattii)* are basidiomycetes that cause systemic fungal infection in animals and humans. These two species have distinct, but also overlapping, environmental niches. *C. gattii* was traditionally considered to only exist in tropical and subtropical regions and was mostly associated with plants such as the *Eucalyptus* species [1]. In contrast, *C. neoformans* has a more global distribution, being isolated mostly in soil contaminated by plant debris and bird droppings. In addition, *C. neoformans* has been isolated from a variety of plant species [2], including indigenous African trees that have been proposed as the origin of *C. neoformans* in Africa [3], suggesting that this species also has an arboreal niche.

The details as to why *Cryptococcus* prefers tree or other environmental niches remain unclear. *Cryptococcus* can complete its sexual cycle by associating with plants, suggesting such association is beneficial for the fungus [4]. Because cryptococcosis is noncommunicable between humans, the initial infection is likely exclusively caused by environmental sources. Basidiospores are thought to be the initial infectious particles inhaled by the human host to cause cryptococcosis, as spores are small enough to lodge into the deep alveoli of the lung and are fully virulent [5], [6]. Hence, mating and recombination of *Cryptococcus* have to occur in nature, as supported by population studies of environmental isolates [7], [8]. However, neither mating nor basidiospores have yet been observed in the environment. The discovery of *Cryptococcus* mating on plants sheds light on the whereabouts of *Cryptococcus* spores in the environment. Recently it was found that inositol secreted from plants stimulates *Cryptococcus* mating [4]. The importance of inositol in the mating of *Schizosaccharomyces pombe* [9] and for fertility of plants and humans has also been reported [10], [11], suggesting a conserved contribution of inositol in sexual reproduction.

There are two main sources from which fungal cells acquire inositol. For one, intracellular glucose can be used to produce inositol in a multiple-step inositol biosynthetic pathway in which the inositol 3-phosphate synthase (Ino1) is the rate-determining enzyme [12]. Inositol can also be imported from the extracellular environment via inositol transporters (*ITR*s). *Cryptococcus* can use inositol both as a carbon source and as a precursor to generate secondary messages that are important for regulating cellular functions and for adapting to environmental signals. Interestingly, in contrast to only one or two inositol transporters present in most fungi, *Cryptococcus* contains an unusually large inositol transporter gene family with over ten members, derived in part from recent gene duplications, suggesting *Cryptococcus* has evolved by associating with the tree niches for inositol utilization [13] (Table 1). Among the transporters, Itr1 and Itr1a are two required for fungal mating [13]. The ability to sense and efficiently acquire inositol from plant surfaces could fuel *Cryptococcus* in its proliferation and sporulation.

**Table 1:** Number of inositol transporter (*ITR*) candidates in fungi.

Fungal species		Group I* *ITR*s	Group II* *ITR*s	Total *ITR*s
Zygomycota	*Rhizopus oryzae*	3	1	4
Basidiomycota	*Ustilago maydis*	2	1	3
	*Cryptococcus neoformans* var. *neoformans* JEC21	8	3	11
	*Cryptococcus neoformans* var. *grubii* H99	7	3	10
	*Cryptococcus gattii* VGI WM276	4	3	7
	*Cryptococcus gattii* VGII R265	3	3	6
	*Tremella mesenterica*	2	1	3
Ascomycota	*Schizosaccharomyces pombe*	2	0	2
	*Coccidioides immitis*	1	1	2
	*Aspergillus fumigatus*	2	3	5
	*Neurospora crassa*	1	1	2
	*Candida albicans*	1	1	2
	*Ashbya gossypii*	1	1	2
	*Saccharomyces cerevisiae*	2	0	2

* *ITR* candidates can be divided into two distinct groups based on overall amino acid sequence similarity. Group I *ITR*s are candidates that are highly similar to the known *ITR*s in *Saccharomyces cerevisiae*; Group II candidates are less conserved but are closely related to known *ITR*s in other fungi.

## Adapting to Trees and Other Niches May Contribute to the Emergence of *Cryptococcus* Virulence in Humans

The progenitor of *Cryptococcus* existed before humans or other warm-blooded mammals populated the world, and plants or plant materials could well represent the original niches for *Cryptococcus*, as suggested by a recent report [3]. The successful transmission from an environmental host to a warm-blood mammalian host defines a precondition for the success of a human pathogen. Mammals developed a sophisticated defense system to ward off the attack of deadly microbes, including physical barriers (high body temperature and epithelial surfaces) and immune response (innate and adaptive immunity). In addition, nutrient limitation is an important restricting factor for the growth of those microbes in vivo. *Cryptococcus* cells grow well at body temperature (37°C), and possess an enlarged polysaccharide capsule and thick melanized cell wall, which enable these cells to resist the hostile host environment. As an intracellular pathogen, the ability of *Cryptococcus* to survive and replicate in macrophages after phagocytosis has been proposed to be a consequence of adaptations that have evolved for protection against environmental predators in nature, like amoebae [14]. By associating with the plant niche, *Cryptococcus* may have developed a complex nutrient-acquisition system to acquire limited nutrients, including inositol, to support its growth and sexual reproduction. This efficient nutrient utilization system could also play an important role in using nutrients in mammalian hosts. It has been shown that enzymes involved in inositol metabolism and inositol sphingolipid biosynthesis are required for the pathogenesis of *C. neoformans* [15].

## Inositol Acquisition May Contribute to *Cryptococcus* CNS Infection

**Figure 1:**
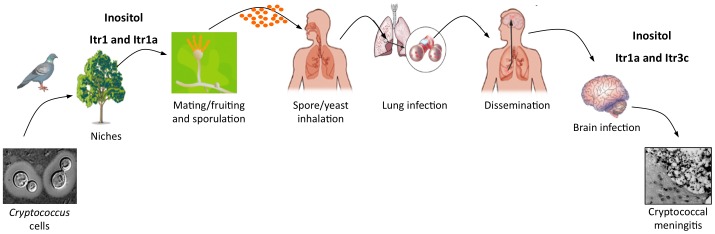
A model of how inositol affects the infection cycle of *C. neoformans*. *Cryptococcus* cells commonly exist in the environment by associating with several niches, including birds, soil, and plants. Inositol is present on plant surfaces and can stimulate fungal mating (including fruiting) to produce infectious spores. Spores inhaled by humans can enter the lungs to cause lung infection. Fungal cells can also be disseminated to the central nervous system (CNS), where abundant inositol is present, and cause fungal meningitis. Inositol can be used as a precursor for both the energy source and the signaling molecule. Part of the model is adapted from Hull and Heitman [6].

The predominant clinical manifestation of cryptococcal infection is the development of fatal meningoencephalitis, especially in people living with AIDS/HIV. The cause of *Cryptococcus* neurotropism remains unclear. Several factors point to inositol as one of the potential host factors promoting the development of cryptococcal meningitis. First, both human and animal brains contain abundant inositol, which plays a critical role in regulating normal neurological responses and psychological feedback [16]. Inositol is a major osmolyte in the human and animal brains and is present in the human cerebellum (5.1 mM) at over 200-fold higher concentrations than are found in plasma (0.02 mM) [16]. Astrocytes that associate with the blood-brain barrier (BBB) contain over 8 mM inositol that can be rapidly released [16]. HIV-infected persons have increased brain inositol levels due to gliosis or increased cell membrane turnover [17]. Second, *Cryptococcus* can utilize inositol as a carbon source, which may provide a growth advantage during brain infection since glucose levels are generally low in brain [18], [19]. Third, *Cryptococcus* can efficiently acquire environmental inositol with its large inositol transporter gene family [4], [13], [20]. Mutants lacking two major fungal inositol transporters, Itr1a and Itr3c, showed attenuated virulence in multiple murine models, indicating that inositol acquisition is required for the *Cryptococcus*–host interaction, particularly during brain infection [4], [13], [20]. Recently, we found that inositol can directly increase the rate of *Cryptococcus* transversal across the human brain macrovascular endothelial cell monolayer in an in vitro model of the BBB, and the inositol effect is fungal inositol transporter–dependent (Liu et al., unpublished). This discovery demonstrates that inositol sensing and utilization could be an important virulence factor for the development of cryptococcal meningitis, which provides a direct biological connection between an environmental adaptation strategy and the emergence of its virulence during human infection (Figure 1).

Despite progress in understanding the role of inositol in *Cryptococcus* pathogenesis, many questions remain unanswered. It remains unclear how inositol promotes fungal cell transversal across the BBB and whether inositol is utilized as a signaling molecule, a carbon source, or both by the fungus during brain infection. It is also unknown whether inositol contributes to the development of capsule structure in *Cryptococcus* and other neurotropic pathogens, since the capsule is one common feature of those pathogens and contributes to their neurotropism. Addressing these questions could lead to a better understanding of the *Cryptococcus* CNS infection.

## Contribution of Inositol to the Virulence of Other Pathogens

Inositol is the precursor for making phosphatidylinositol (PI) and is essential for cellular structure and regulation of intracellular signaling in all eukaryotes. The role of inositol acquisition in the development of virulence has been studied in a variety of fungi, protozoa, and certain eubacteria [12]. Similar to *Cryptococcus*, the yeast pathogens *Candida albicans* and *Candida glabrata (C. glabrata)* can acquire inositol through both de novo biosynthesis pathways and import via inositol transporters. Blocking either pathway does not affect fungal infection, but deleting both pathways is lethal, suggesting inositol acquisition is essential for *Candida* survival and either pathway is sufficient to support fungal growth and full virulence [21]. The inositol regulon is wired differently in *C. albicans* compared to the one in *C. glabrata*, suggesting complex inositol regulatory systems in different fungi [22].

Besides fungi, inositol also plays a role in pathogenicity of other parasitic microorganisms [12]. Interestingly, although parasites such as *Trypanosoma brucei* and *Leishmania mexicana* and mycobacteria such as *Mycobacterium tuberculosis* can both synthesize and import inositol, blocking inositol biosynthesis leads to growth defect and virulence attenuation, indicating inositol uptake itself is not sufficient [23], [24]. Inositol synthesized in cells has been suggested to be the source of PI used for GPI anchor assembly, which may explain the importance of inositol biosynthesis despite the ability of pathogens to import inositol.

## Other Adaption Strategies Associated with *Cryptococcus*

Besides utilizing plants as one niche, *Cryptococcus* cells often associate with certain amoeba species in which the yeast cells can be taken up but survive inside the amoebae, a phenomenon similar to *Cryptococcus*–macrophage interactions. The interaction between *Cryptococcus* and amoebae has been shown to increase the resistance of *Cryptococcus* to phagocytosis during its infection in lung, suggesting that selective pressures placed by amoebae on *Cryptococcus* contribute to the maintenance of fungal virulence in animal hosts [14]. In addition, *Cryptococcus* cells can increase ploidy and significantly enlarge in cell size in vivo as a way of protecting yeast cells from phagocytosis [25]. Nitrogen-rich pigeon guano is another primary ecological niche of *C. neoformans*. Media made of pigeon guano has been shown to stimulate mating of *C. neoformans* but not *C. gattii* [26]. The availability of nitrogen, such as uric acid, has been shown to play a role in *Cryptococcus* virulence [27]. A recent study demonstrated a nitrogen-metabolite repression process to regulate the nitrogen acquisition [28]. Thus, understanding the environmental niches of a particular human pathogen can be very helpful in understanding its disease mechanism. In addition, the adaption of a pathogen to new environmental niches could result in the emergence of new virulence traits. The perfect example is the outbreak of cryptococcosis in otherwise healthy people caused by *C. gattii* in western North America where *Eucalyptus* trees do not exist and it is not a tropical climate. The most common *C. gattii* strain (VGIIa) showed higher proliferation rates in macrophages than other *C. gattii* isolates from around the world: an indication of the emergence of virulence since proliferation rate is correlated with fungal virulence [29]. The emergence of disease caused by *C. gattii* in immunocompetent individuals in temperate Vancouver Island, Canada and its expansion in western North America suggests an evolution of host range, geographic location, and virulence of this pathogen [30].
